# Molecular detection and identification of three intracellular parasites of retail mutton products in Beijing, China

**DOI:** 10.3389/fvets.2022.1018788

**Published:** 2022-09-30

**Authors:** Zifu Zhu, Yajie Chen, Xu Yang, Lifang Wang, Qun Liu, Jing Liu

**Affiliations:** ^1^National Animal Protozoa Laboratory, College of Veterinary Medicine, China Agricultural University, Beijing, China; ^2^Key Laboratory of Animal Epidemiology of the Ministry of Agriculture, College of Veterinary Medicine, China Agricultural University, Beijing, China

**Keywords:** sheep, *Sarcocystis* spp., *N. caninum*, *T. gondii*, prevalence

## Abstract

*Sarcocystis* spp., *Neospora caninum* and *Toxoplasma gondii* are globally ubiquitous pathogens, and domestic sheep are considered to be one of the intermediate hosts. 83 myocardial samples of sheep were collected from 12 retail stores in Beijing, China. *Sarcocystis* spp., *N. caninum* and *T. gondii* were identified by PCR amplification of the 18S rRNA gene, Nc-5 gene and 529bp DNA fragment with a prevalence of 86.7% (95% CI: 77.5–93.2) and 43.4% (95% CI: 32.5–54.7) for *Sarcocystis* spp. and *N. caninum* infections, respectively, and no *T. gondii* was detected. The co-infection prevalence of *Sarcocystis* and *N. caninum* was 38.6% (95% CI: 28.1–49.9). Two *Sarcocystis* species were subtyped by analyzing 18SrRNA sequences and were identified as *Sarcocystis tenella* and *Sarcocystis arieticanis*. The prevalence of *S. tenella* and *S. arieticanis* infections was 84.3% (95% CI: 74.7–91.4) and 56.6% (95% CI: 45.3–67.5), respectively. This study shows that sheep have a high risk of infection with *Sarcocystis* and *N. caninum*, suggests that effective prevention measures are needed to avoid the spread of these parasites in sheep. Toxoplasmosis in sheep poses a threat to human and animal health and requires monitoring and preventing continuously.

## Introduction

Toxoplasmosis, neosporosis and sarcocystosis are parasitic diseases caused by *Toxoplasma gondii, Neospora caninum* and *Sarcocystis* spp., respectively (1–3). These diseases have a wide geographic distribution and the ability to infect warm-blooded animals. Toxoplasmosis is an important zoonotic disease that not only causes severe reproductive and economic losses, but also poses a great threat to public health (2). Humans are primarily infected through ingestion of raw or undercooked meat containing tissue cysts or food contaminated with oocysts that have been disseminated by cats or other felids (4). A series of investigations of *T. gondii* infections in farm animals have confirmed that the meat of sheep is one of the most important sources of infection, with seroprevalence of *T. gondii* ranging from 1.2 to 39.1% in sheep in China (5).

*N. caninum* has been identified as an important cause of reproductive failure in cattle and small ruminants (1, 6). Although antibodies against *N. caninum* have been reported in human serum samples, its zoonotic potential has not been confirmed (7). In China, molecular survey on *N. caninum* in Chinese sheep is rare and focuses mainly on serological investigations, with seroprevalence of *N. caninum* infection among Chinese sheep ranging from 7.32 to 57.25% (8–11).

In China, three validated species of *Sarcocystis* have been described in sheep: *S. tenella, S. arieticanis*, and *S. gigantea* (12). *S. tenella* and *S. arieticanis* are pathogenic and can lead to abortion, neurological symptoms, and even death in the early stages of infection and chronic disease in the late stages of infection (3). *Sarcocystis* infection in sheep has been reported in several studies with a prevalence of 7.74–100% (12).

Under natural conditions, sheep may be infected with these protozoa at any time (13). However, no studies have investigated the co-infection prevalence of these three protozoa among sheep in the Beijing area. Therefore, the aims of the present study were to investigate the prevalence of *Sarcocystis* spp., *N. caninum* and *T. gondii* in retail sheep hearts from Beijing, China.

## Materials and methods

### Sample collection and processing

A total of 83 sheep hearts (all sheep were about 2 years old) were collected between August 2020 to January 2021 from 12 meat retail stores in Beijing, China. A minimum of five to a maximum of ten hearts were collected each week. Samples collected were marked with date and store location and transferred to the laboratory in cool conditions. Samples were analyzed for the presence of *Sarcocystis* cysts by gross inspection, examination of unstained squash preparations, and tissue digestion to detect the release of bradyzoites from tissue cysts. All operations were completed within 3 days to ensure freshness of the samples. Digestive fluids or muscle tissues were submitted to PCR for DNA of *Sarcocystis* spp., *N. caninum* and *T. gondii* detection.

### Detection of tissue cysts and bradyzoites

Muscle connective tissue and fat were removed from the sample, and three rice-sized portions of muscle tissue were randomly selected from each sample along the direction of the muscle fibers and pressed between two glass slides to make them thin. Then, the tissue was observed under a 10× magnification microscope. The remaining tissue was digested with HCl-pepsin solution (14).

Each sample (50 g) was digested in HCl-pepsin solution, and then 100 μL of the digestive fluid was taken for detection of bradyzoites under light microscopy (14). Protozoan bradyzoites were purified using the Percoll density gradient centrifugation method (14). The purified bradyzoites were preserved at −20°C for molecular identification.

### DNA extraction and PCR amplification

Genomic DNA was extracted from purified bradyzoites and/or tissue homogenate of each sample following the instructions of the Genomic DNA Extraction Kit (Aidlab Biotech, Beijing, China). The extracted DNA was stored at −20°C until PCR amplification. Identification of *T. gondii, N. caninum* and *Sarcocystis* spp. was performed by PCR using primers for species-specific genes. The primer sequences are shown in Table 1. 20 μL of PCR mix was used for each amplification: 10 μL of 2 × M5 HiPer plus Taq HiFi PCR mix (Mei5 Biotechnology, Co., Ltd, Beijing, China), 1 μM of each primer (forward and reverse), 6 μL deionized water, 2 μL of genomic DNA for the primary PCR template, or 2 μL of diluted primary amplification products used for the secondary PCR templates. PCR primers are shown in Table 1. PCR was performed using the T100^TM^ Thermal Cycler (Bio-Rad). Each PCR assay had a negative control (deionized water) and a positive control (*T. gondii* RH standard strain, *N. caninum* Nc-1 standard strain, previously characterized as a positive control for *S. tenella* or *S. arieticanis* DNA, respectively). The PCR products were visualized by 1.5 g/L agarose gel electrophoresis. Each sample was subjected under three PCR replications.

**Table 1:** Information of the primers used in PCR detection of the three species of parasites.

**Species**	**Targeted gene**	**Primer (5^′^−3^′^)**	**Fragment (bp)**	**Annealing (°C)**	**Amplification method**	**References**
*S. tenella*	18SrRNA	external forward (ST1: 5'-GGA TCG CAT TAT GGT CAT-3')	1563	57	Nest PCR	(15)
		external reverse (AP2: 5'-CCC GGG ATC CAA GCT TGA TCC TTC TGC AGG TTC ACC TAC-3')				
		Internal forward (8: 5'-TTT GAC TCA ACA CGG G A-3')	530	59		
		Internal reverse (ST3: 5'-CGT TGC CGC GCG TTA A-3')				
*S. arieticanis*	18SrRNA	external forward (STA: 5'-TTT CGC AAG GAA GAG GA-3')	1598	57	Nest PCR	(15)
		external reverse (SA2: 5'-TGA AAC GGC GCG TAG A-3')				
		Internal forward (2: 5'-AGG GTT CGA TTC CGG AG-3')	375	59		
		Internal reverse (SA1: 5'-GCG G GA AGA GGA GAA T-3')				
*Sarcocystis* spp.	18SrRNA	Forward (Sar-F1: 5'-GCACTTGA T GA ATTC T GGCA-3')	600	55	Standard PCR	(16)
		Reverse (Sar-R1: 5'-CACCA CC C AT AG AA TCAAG−3')				
*N. caninum*	Nc-5 gene	Forward (Np21: 5'-GTGCGTCC AATCCTGTAA-3')	328	58	Standard PCR	(17)
		Reverse (Np6: 5'-CAGTCAACC T AC GT CT T C-3')				
*T. gondii*	529bp DNA fragment	TOX4 (5'-CGCTGCAGGGAGGA AG AC G A A AGTTG-3')	529	55	Standard PCR	(18)

### Nucleotide sequencing and analysis

The positive PCR products of the target genes were direct sequenced bidirectionally by a sanger sequencing in a commercial sequencing company (Ruibiotech, Beijing, China). The obtained nucleotide sequences were aligned with reference sequences on the National Center for Biotechnology Information (https://blast.ncbi.nlm.nih.gov/Blast.cgi).

### Statistical analysis

A Chi-square test was applied to compare the prevalence of *Sarcocystis* spp., *N. caninum* and *T. gondii* in sheep using GraphPad Prism 8.0 software. Statistical significance was set at *p* < *0.05*.

## Results

### Prevalence rates of three intracellular parasites in retail sheep

**Figure 1:**
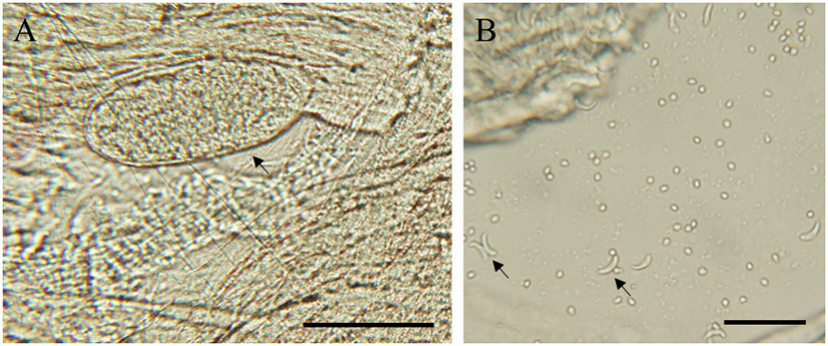
Morphological characteristics of *S. tenella* in the myocardium of sheep. **(A)** light micrograph of a sarcocyst (arrow), unstained; **(B)** light micrograph of bradyzoites (arrow) in pepsin-digestion liquid; unstained. Bar = 50 μm.

The sample were considered positive if at least one typical cyst or bradyzoite was observed. In this study, no visible cysts were observed in 83 sheep heart samples. Light microscopy revealed *Sarcocystis* cysts that were spindle-shaped or oval in shape, parallel to the muscle fibers, and contained large numbers of bradyzoites (Figure 1A). Besides, all samples were digested in HCl-pepsin solution to detect bradyzoites released from muscle tissue (Figure 1B). All samples confirmed by microscopy were tested positive for *Sarcocystis* based on the 18SrRNA gene. In summary, the prevalence of muscle squashing microscopic observation, pepsin digestion examination and PCR detection was 26.5% (95% CI: 17.4–37.3), 77.1% (95% CI: 66.6–85.6) and 86.7% (95% CI: 77.5–93.2), respectively (Table 2).

**Table 2:** Prevalence of *Sarcocystis* spp. infection among sheep according to the diagnostic method.

**Diagnostic method**	**Unstained squashes**	**Pepsin digestion**	**PCR**
Prevalence (infected/examined)	26.5% (22/83)	77.1% (64/83)	86.7% (72/83)

Of the 83 muscle samples collected from Beijing, 36 (43.4%, 95% CI: 32.5–54.7) and 0 (0.0%, 95% CI: 0.0–4.3) tested positive for *N. caninum* and *T. gondii* based on the Nc-5 gene sequence and the *T. gondii* 529 bp DNA fragment, respectively. The co-infection prevalence of *Sarcocystis* and *N. caninum* was 38.6% (95% CI: 28.1–49.9; Table 3).

**Table 3:** Prevalence of *Sarcocystis, N. caninum* and *T. gondii* infection among sheep.

**No. samples**	***Sarcocystis*** **species**				***N. caninum***	***T. gondii***	***Sarcocystis + N. caninum***
	***S. tenella***	***S. arieticanis***	***S. tenella* and/or *S. arieticanis***	***Sarcocystis* spp**.			
83	84.3% (70/83)	56.6% (47/83)	86.7% (72/83)	85.5% (71/83)	43.4% (36/83)	0 (0/83)	38.6% (32/83)

### Molecular identification of *Sarcocystis* spp. and *N. caninum*

From the 18SrRNA gene of *S. tenella* and *S. arieticanis*, 70 (84.3%, 95% CI: 74.7–91.4) out of 83 muscle specimens tested positive for *S. tenella* and 47 (56.6%, 95% CI: 45.3–67.5) tested positive for *S. arieticanis*. *S. tenella* was the most prevalent identified *Sarcocystis* species. The co-infection prevalence of *S. tenella* and *S. arieticanis* was 54.2% (95% CI: 42.9–65.2; Table 3). After sequencing of PCR products, the nucleotide sequence of the 18SrRNA gene fragment of *S. tenella* (*n* = 67) from the Beijing sheep samples was identical to isolates from the Chinese domestic sheep (MF039329), and the three *S. tenella* sequences showed 99.81% homology with MF039329 and one substitution at nucleotide position 1309 (A→ G). The *S. arieticanis* sequence (*n* = 47) was identical to isolates from the Chinese domestic sheep (MF039331). The *N. caninum* sequence (*n* = 36) was identical to isolates from aborted bovine fetuses in China (EF581827), followed by *N. caninum* (EF463099) from Polish cattle (98.51% identity), and *N. caninum* (MT340527) from pigs in China (97.01% identity).

## Discussion

The sheep industry is an important part of China's animal husbandry, with mutton being a major ingredient in hot pots and barbecues, and consumption of undercooked meat containing *T. gondii* tissue cysts poses a health risk. Sheep is also the intermediate host of *N. caninum* and *Sarcocystis* spp., and large numbers of parasitic tissue cysts in the muscle can reduce meat quality and compromise food hygiene (13). Mutton fed to dogs and cats can also lead to infection and parasite transmission in pets and economic animals. However, molecular survey on sheep infected with *T. gondii, N. caninum* and *Sarcocystis* spp. are limited. Here, we investigated the molecular prevalence of *Sarcocystis, N. caninum* and *T. gondii* in sheep intended for human consumption in Beijing, China. The molecular prevalence of *Sarcocystis* spp. and *N. caninum* was 86.7 and 43.4%, respectively. *T. gondii* was not observed. These results highlight the public health risks of *Sarcocystis* spp. and *N. caninum* as well as the prevalence of sarcocystosis and neosporosis in sheep farms in Beijing, China.

*Sarcocystis* is usually detected by muscle squashing microscopic observation and histological examination. Since the occurrence of sarcocysts detection is random, the detection rate examination of unstained squash preparations will be slightly lower than the true value. Digestion of host tissue with HCl-pepsin solution has been reported to be the most sensitive method for detecting mild infections of *Sarcocystis* (3). However, these methods do not identify *Sarcocystis* species. Heckeroth et al. established species-specific nested PCR assays based on the unique 18SrRNA gene sequences of *S. tenella* and *S. arieticanis* to diagnose and differentiate between *S. tenella* and *S. arieticanis* infections in sheep (15). In comparison with other methods, the HCl-pepsin digestion method combined with nested PCR assays showed a higher sensitivity for the detection of *Sarcocystis*. Hence, the 86.7% prevalence of *Sarcocystis* infection in sheep found in this study may be close to the true value, and the identification of *S. tenella* and *S. arieticanis* confirms that this experimental design can accurately identify multiple *Sarcocystis* infections in actual samples.

The prevalence of *Sarcocystis* found in this study was higher than that in Henan, Xinjiang and Qinghai, but lower than that in Yunnan (19–21). Combined with these studies on *Sarcocystis* infection, it is implied that *Sarcocystis* spp. may be widely spread among sheep in China. The prevalence of *Sarcocystis* spp. in sheep varies in different regions of the world. The prevalence rate of *Sarcocystis* in other countries was reported to be 63.83% (95% CI: 45.84–80.01) in Iran (22), 96.9% in Mongolia (23), 95.8% in Brazil (24). *Sarcocystis* can cause weight loss, abortion, premature birth, and even death in sheep (3). Animals usually infected by ingesting food or drinking water contaminated with *Sarcocystis* sporocysts (3). Although sheep sarcocystiosis is not zoonotic, *Sarcocystis* spp. possess the powerful sar-cocystin neurotoxin, *Sarcocystis* spp. infection is a cause of condemnation of adult sheep meat (25). Therefore, it is necessary to enhance the husbandry management of sheep to prevent and control *Sarcocystis* infections.

In this study, the detection rate of *N. caninum* was 43.4% (95% CI: 32.5–54.7), which is significantly higher than that of Central China (7.32%) (26) and Southwest China (8.55%) (11). Compared to other studies in the world, it was higher than the rates in Iran (6.7%) (27), North Africa (10.6 ± 4.3%) (28), but lower than those observed in São Paulo, Brazil (59.23%) (29) and the state of Pernambuco, Brazil (64.2%) (30). Although the economic, clinical, and epidemiological importance of *N. caninum* infection in sheep remains uncertain, studies have reported that *N. caninum* can cause abortion, birth of weak lambs, suggested an association between *N. caninum* infection and reproductive losses in sheep (31, 32). In addition, antibodies to *N. caninum* have been reported in human serum, but the parasite has not been detected in human tissues, the zoonotic potential is uncertain (7).

Variations in the prevalence of *N. caninum* in different regions may be related to sheep breeds, age of test samples, detection methods, sample sizes, as well as farm hygiene management and animal health status. Furthermore, these sheep are free-ranging in rural areas, where most farmers usually keep dogs to protect the sheep sheds. Dogs are the final hosts of *S. tenella, S. arieticanis* and *N. caninum*, and sheep can be infected by ingesting sporulated oocysts found in the feces of infected dogs in contaminated food or drinking water. Therefore, the presence of dogs is also a potential factor in the high prevalence of *S. tenella* and *N. caninum*.

In this study, the prevalence of *T. gondii* infection was 0.0% (95% CI: 0.0–4.3), which was significantly lower than the prevalence of *T. gondii* in sheep worldwide (14.7%, 95% CI: 0–57) (33). *T. gondii* tissue cysts have been usually reported to parasitize the muscles and CNS. In sheep, samples pooled within the animal resulted in a significantly higher prevalence compared to single organ samples (33). Therefore, the low detection rate in this study may be due to insufficient sample size, low level of infection, and sample types. *T. gondii* is an important zoonotic species that is usually found in the muscles, viscera, and blood of warm-blooded animals (2). Carnivorous animals and humans can be infected with *T. gondii* via the consumption of rarely cooked meat (mutton, beef, or viscera) containing tissue cysts, with livestock considered to be the major source of human infection (2). Mutton has very rich nutritional value and is popular in China. In this case, humans are at high risk for *T. gondii* infection through ingestion of undercooked or raw meat of infected sheep, which may contain *T. gondii* cysts. Although no *T. gondii* infection was detected in this study, the pooled prevalence of *T. gondii* in China is 8.5% (95% CI: 6.5–10.9) (34), it is necessary to monitor regularly *T. gondii* infection in sheep.

In conclusion, this is the first molecular survey and characterization of *Sarcocystis* spp., *N. caninum* and *T. gondii* infections among sheep in Beijing, China. This study shows that *S. tenella, S. arieticanis* and *N. caninum* are highly prevalent in Beijing sheeps. The high prevalence of *Sarcocystis* spp. and *N. caninum* in sheep indicates the need for effective prevention measures, as improve breeding management in sheep, cats and dogs, to avoid the spread of these parasites in the sheep industry and in public health. These results will provide a reference basis for further research and control of the three intracellular parasites among sheep in China.

## Data availability statement

The raw data supporting the conclusions of this article will be made available by the authors, without undue reservation.

## Ethics statement

The animal study was reviewed and approved by the Institutional Animal Care and Use Committee of China Agricultural University (Approval No. AW71211202-2-1).

## Author contributions

ZZ: data curation, formal analysis, investigation, project administration, and writing-original draft. YC: investigation and methodology. XY: investigation and software. LW: methodology. QL: data curation, methodology, conceptualization, and supervision. JL: conceptualization, funding acquisition, resources, supervision, validation, and writing—review and editing. All authors contributed to the article and approved the submitted version.

## Funding

This work was supported by the National Natural Science Foundation of China (31972700) and the Beijing Municipal Natural Science Foundation (62732622).

## Conflict of interest

The authors declare that the research was conducted in the absence of any commercial or financial relationships that could be construed as a potential conflict of interest.

## Publisher's note

All claims expressed in this article are solely those of the authors and do not necessarily represent those of their affiliated organizations, or those of the publisher, the editors and the reviewers. Any product that may be evaluated in this article, or claim that may be made by its manufacturer, is not guaranteed or endorsed by the publisher.
